# Microbiota-directed biotherapeutics: considerations for quality and functional assessment

**DOI:** 10.1080/19490976.2023.2186671

**Published:** 2023-03-10

**Authors:** Emily Ef Fekete, Daniel Figeys, Xu Zhang

**Affiliations:** a Regulatory Research Division, Centre for Oncology, Radiopharmaceuticals and Research, Biologic and Radiopharmaceutical Drugs Directorate, Health Products and Food Branch, Health Canada, Ottawa, Canada; b School of Pharmaceutical Sciences, Faculty of Medicine, University of Ottawa, Ottawa, Canada; c Ottawa Institute of Systems Biology and Department of Biochemistry, Microbiology and Immunology, Faculty of Medicine, University of Ottawa, Ottawa, Canada

**Keywords:** Biotherapeutics, metaproteomics, microbiome assay, microbiota, multi-omics

## Abstract

Mounting evidence points to causative or correlative roles of gut microbiome in the development of a myriad of diseases ranging from gastrointestinal diseases, metabolic diseases to neurological disorders and cancers. Consequently, efforts have been made to develop and apply therapeutics targeting the human microbiome, in particular the gut microbiota, for treating diseases and maintaining wellness. Here we summarize the current development of gut microbiota-directed therapeutics with a focus on novel biotherapeutics, elaborate the need of advanced -omics approaches for evaluating the microbiota-type biotherapeutics, and discuss the clinical and regulatory challenges. We also discuss the development and potential application of *ex vivo* microbiome assays and *in vitro* intestinal cellular models in this context. Altogether, this review aims to provide a broad view of promises and challenges of the emerging field of microbiome-directed human healthcare.

## 1. Introduction

The human microbiota is a collection of microorganisms that inhabit the human body with the majority being in the gastrointestinal (GI) tract. In the past decades, extensive efforts have been made to catalog the microbial species, genes and genomes at different sites of the human body. These efforts have led to the identification of > 4000 microbial species, > 170 million genes, and>20,000 genomes in the human GI tract alone.^1–3^ Moreover, accumulating evidence also shows that the human GI tract is home to diverse archaea, fungi, viruses and parasites, in addition to bacteria.^4–7^ An increasing number of studies have shown that the dysbiosis of human gut microbiota was associated with a variety of diseases, including gastrointestinal diseases, metabolic diseases, neurological disorders, and impaired patient response to immunotherapy for cancers.^8–10^

Uncovering the critical roles of the microbiome in human health is one of the most striking advances in the field of biomedicine in the past decade. Nowadays, the human is considered as a superorganism, consisting of both the human cells and resident microbes that are present at almost all surfaces of the human body.^11^ The microbiome and the host respond to external perturbations, including drugs and nutrients, together to influence human health.^12^ Therefore, the human microbiome is emerging as an important target for disease management, which can be partly due to its advantage of high genomic plasticity (or druggable genomes) when compared to human genome itself.^13^ Various types of microbiota-directed therapeutics have been developed in the past few years, ranging from simple chemical compounds to complex microbial communities. Some of them have already been under evaluations in late phase clinical trials with a promise to be accessible to patients.

Two traditional and well-established therapeutic approaches targeting the gut microbiome include dietary intervention and xenobiotics. Dietary regimes have been widely studied and are known to have drastic impacts on gut microbiota composition, favoring the growth of commensal beneficial bacteria while inhibiting harmful bacteria.^14,15^ Xenobiotics are chemical substances which may be capable of modulating gut microbiota. Recent studies showed that >20% of Food and Drug Administration (FDA)-approved drugs were able to influence the growth of gut bacteria,^16^ and some microbes can metabolize or accumulate drugs to affect the drug efficacy and toxicity.^17,18^ Repurposing these clinically used drugs for microbiome-targeted therapy^19^ and the discovery of new precise microbiota-editing compounds, such as tungstate^20^ is now an important topic of biomedical research. In addition to diet and xenobiotics, there is accumulating evidence to highlight the advantages of implementing microbiome science and the use of biological therapeutics (or biotherapeutics) for personalized and precision medicine. Microbiota-directed biological therapeutics include but are not limited to conventional biologics such as antimicrobial peptides, live biotherapeutic products (LBPs) such as fecal microbiota therapy (FMT), and next-generation probiotics. In particular, FMT has a long history of clinical application and was used as a standard-of-care for recurrent *Clostridioides difficile* infection (rCDI) in many countries.^21–23^ In limited studies, FMT has also been shown to improve anti-programmed cell death protein 1 (anti-PD-1) response in immunotherapy-refractory cancer patients,^24,25^ indicating that targeting the microbiome is a promising way to improve the efficacy of precision cancer therapy.

While the microbiota-directed biotherapeutics are promising, it remains challenging to perform efficient quality assessment. Most current platforms for drug development and regulation are established for chemical xenobiotics that target a single pathogenic bacterium or specific gene of the host. The characterization of the complex microbiomes requires more advanced bioanalytical techniques, such as -omics and meta-omics. The assays for evaluating microbiota functionality require unique facilities and platforms that enable growth and maintenance of a wide range of complex microbiota. In this review, we will focus on the current development of novel gut microbiota-directed biotherapeutics. We will elaborate the benefit and application of multi-omics, *ex vivo* microbiome assays and *in vitro* intestinal cellular models for evaluating microbiome-targeted biotherapeutics. We will also discuss the clinical and regulatory challenges of applying microbiome therapeutics for human healthcare.

## 2. Novel microbiota-directed biotherapeutics

### 2.1 Fecal matter derived biotherapeutics

Transferring raw fecal material, namely FMT, from a donor into CDI patients has been shown to successfully prevent and/or treat the recurrent *C. difficile* infection.^26,27^ FMT has also been evaluated in clinical trials for their efficacy on inflammatory bowel disease (IBD), irritable bowel syndrome (IBS), alcohol use disorders, obesity, as well as their efficacies in overcoming resistance to anti-PD1 immunotherapy in cancer patients.^24,25,28–31^ More recently, FMT was demonstrated to be beneficial for the management of aging and neurological diseases, such as Alzheimer’s disease and autism-spectrum disorders (ASD) in both humans and animal models,^32–35^ adding to the accumulating evidence on the bidirectional roles of microbiota in gut-brain axis. Fecal microbiota transfer from young to aged mice was reported to reverse hallmarks of aging in the gut, eye, and brain tissues in aged mice.^36^ Most current FMTs were performed using either fresh or frozen fecal matter with colonoscopes or enema, however this is challenging due to the difficulty in getting fresh feces at the time of colonoscopy or enema, the limited access to colonoscopic equipment, as well as the patient non-acceptability to fresh stools as therapeutics. Oral capsule of lyophilized or fresh fecal matter is an alternative dose form that is convenient to administer and has been shown to have equivalent clinical efficacies in treating ulcerative colitis and CDI.^37,38^ Despite the therapeutic promises of FMT, the use of donor feces as a therapeutic agent has its own unique set of risks. Unlike typical chemical-based drugs the variability between donor samples does not only result in variable treatment efficacy, but can also leave the patient vulnerable to pathogen transfer and acquisition of other complications.^39^ Therefore, each donation requires rigorous screening and quality assessment to ensure recipient safety. However, the list of screening pathogens is not always complete, which leads to the risk of safety issues with a prime example being death of a patient after receiving an FMT containing a multi-drug-resistant strain of *Escherichia coli* in 2019.^39^ This emphasizes the need for better safety assurance strategies in FMT biotherapeutics as well as motivates the search for alternative biotherapeutics, such as synthetic microbial communities, next-generation probiotics, and phage therapies (Table 1).

**Table 1.** Advantages and limitations of different microbiota-directed biotherapeutics for translational applications.

Therapeutics		Clinical/Regulatory Advantages	Clinical/Regulatory Limitations
Fecal Matter Derived Biotherapeutics	Fecal Microbiota Transplant (FMT)	Contains full range of microorganisms present in the donor’s feces Clinically evident efficacy at restoring microbiome balance and treating various diseases	Extremely high complexity and not fully characterized Difficulty in recruiting donors and donor screening High inter-donor variability of fecal materials High manufacturing process-introduced variability Reduced patient access due to the need of colonoscopic equipment (if fresh or frozen dosage forms are used) Unknown mechanisms of actions, making it difficult to identify critical quality attributes and develop potency assays Underlying risk of transmission of undetected or emerging pathogens Filtered or processed fecal transplants may be less effective as they exclude some substances in feces that may be of therapeutic importance
	Fecal Filtrate Transplantation (FFT)	Reduced risk of pathogen transmission to patient recipients Less complexity without cellular organisms	
	Processed or modified fecal matter transfer	Reduced risk of pathogen transmission to patient recipients Enrichment/depletion of certain bacterial subgroups	
Synthetic Microbial Community		Reduced reliance on donors and donor samples Reduced risk for harmful pathogens to infect patient recipients Known microbial contents and compositions for quality assurance and assessment	Not full representative of the entire fecal microbiota Challenging to select and determine the microbial combinations Generation, culture, co-culture, and preservation of these communities are very difficult and exact reproducibility is challenging Possible legal issues surrounding ownership of synthetic microbial communities which could impact development and commercialization
Next-Generation Probiotics		Ability for pure culture Strains used can be well classified and defined Relatively straightforward generation, culture, and preservation of individual probiotic strain	Not a replacement or substitute for fecal microbiota therapy, with not fully revealed ecological effects on gut microbial community Limited regulatory guidelines due to classification as food supplement rather than drug Unknowns regarding shelf-life for probiotic potency and the effect of probiotic viability Conflicting observations may exist and have some clinical scenarios with unwanted side effects or worse outcomes
Engineered Bacterial Strains		Ability for function specific targeting/individualization Known mechanisms of action Easy to determine critical quality attributes and develop potency assays Derived from well-characterized and widely used strains, and thereby easy for manufacturing	Not a replacement or substitute for fecal microbiota therapy, with not fully revealed ecological effects on gut microbial community Limited regulatory guidelines due to the involvement of genes or genetic elements not normally found in the human body which may raise safety concerns Unknowns regarding *in vivo* and in human efficacy
Bacteriophages		High specificity, genomic plasticity Auto-dosing capacity, potential for single-dose and low-dose use thanks to multiplication rates Minimal disruption of normal microbiota Versatile dosage forms and can be applied as cocktails Low inherent toxicities Can be well classified and defined	Narrow host range can limit treatment efficacy Bacterial development of resistance Potential immunogenicity in humans Limited regulatory guidelines for quality assessment and manufacturing Unknowns regarding shelf-life for phage potency and stability Lytic to temperate phages conversion Potential toxin-carrying and to transfer genes between bacteria through transduction
Postbiotics	Short-chain fatty acids (SCFAs) Exopolysaccharides (EPS) Extracellular vesicles (EVs)	Do not contain live microorganisms and may be compatible with current manufacturing and regulatory frameworks May be better tolerated than live biotherapeutics Can be generated as chemically defined substances, such as SCFAs	Not fully characterized mechanisms of action, making it difficult to identify critical quality attributes and develop potency assays Can be complex mixture of secreted metabolites with high batch-to-batch variations May be less effective at restoring the balance of the microbiome
Antimicrobial Peptides		Broad-spectrum activity (can work against bacteria, viruses, or fungi) Generally resistant to bacterial resistance Known chemical structure and compatible with current manufacturing and regulatory frameworks Have anti-inflammatory and immune modulatory effects	Systemic and local toxicity Shelf-life/degradation from susceptibility to proteolysis Activity sensitive to physiochemical conditions (salt, serum, pH, etc.) Sensitization and allergy after repeated application

Fecal filtrate transplantation (FFT) represents another promising alternative for FMT. Ott et al. reported that transfer of sterile filtrates from donor stool, rather than fecal microbiota, can be sufficient to restore normal stool habits and eliminate symptoms of CDI in a small patient cohort.^40^ In FFT, all living bacteria are removed, which reduced the risk for harmful bacteria to infect patient recipients, with resulting therapeutic effects induced by bacterial metabolites, proteins, DNA, or antimicrobial compounds remaining in the filtrate.^40^ More recently, FFT has been reported to efficiently prevent necrotizing enterocolitis without detectible side effects.^41^ One proposed mechanism of action for FFT treatment of necrotizing enterocolitis was the increase of phages located in the mucus layer of the gut, which resulted in reduced relative abundance of certain bacteria close to the mucosa.^41^ These findings indicate that the microbial metabolites, secreted proteins/peptides or viral particles such as phages can directly or indirectly alter the composition of patient gut microbiomes and might be a key contributor for mediating beneficial effects of FMT.

Processed or modified fecal matter transfer is another proxy of FMT. Researchers at Seres Therapeutics Inc. (MA, US) developed a manufacturing procedure to enrich spore-forming Firmicutes species while eliminating Gram-negative pathogens and debris from fecal matter to generate an investigational microbiome drug, termed SER-109.^42^ SER-109 is an oral microbiome therapy that is formulated as capsules and consisted of a consortium of bacterial spores, meant to metabolically out-compete and/or alter bile-acid profiles to reestablish colonization resistance to *C. difficile*.^43^ A phase 3, double-blind, randomized, placebo-controlled clinical trial demonstrated that SER-109 was able to reduce the risk of recurrent CDI and showed a similar safety profile to that of the placebo.^43^ SER-109 therefore demonstrates the therapeutic use of processed fecal matter, in this case the administration of bacterial spores from a subset of the bacteria found in donor fecal matter to mitigate risk of transmitting infectious agents through fecal matter. It is worth noting that the processed fecal matter is not representative of the entire microbiota and its efficacy will be more likely disease- and/or population-specific depending on the mechanisms of actions.

### 2.2 Synthetic microbial community

The human gut microbiome is highly individualized and extremely complex, which makes it difficult to perform efficient quality control and characterization of the products for transplanting raw fecal matter. To address this issue, synthetic microbial communities that can represent the fecal microbiota have been designed and generated. Petrof et al. selected 33 bacterial isolates from a healthy donor to generate a synthetic microbial community, termed Microbial Ecosystem Therapeutic 1 (MET-1), which was used as a stool substitute formulation and successfully cured antibiotic-resistant *C. difficile* induced colitis in two patients.^44^ Further, Kao et al. developed MET-2, a prototype microbiome therapy consisting of 40 lyophilized bacterial species, which was shown to be safe, efficacious, and well tolerated among patients with recurrent CDI.^45^ More recently, Cheng et al. designed a more complex defined community consisting of 119 species (termed hCom2).^46^ hCom2-associated gnotobiotic mice were phenotypically similar to those associated with a human fecal microbiota and exhibited efficient colonization resistance against pathogenic *E. coli*.

Many studies have shown the therapeutic benefits and promise of various synthetic microbial communities, but they are not without their own unique set of challenges. The first challenge of generating a synthetic microbial community is in the selection of which bacterial species to include and whether the combination should be based on phylogeny, metabolic profile, or function. There is estimated to be hundreds of bacterial species in an individual’s gut microbiome, as well as hundreds of species of other microorganisms (i.e. viruses, archaea). With the large -omics datasets being generated for patient and healthy individual’s microbiomes and the growing available computing power, mathematical models can be applied to design optimal synthetic microbial communities for therapy. An example of this include mathematical modeling of microbiota data from patients and mice with varied *C. difficile* susceptibility to rationally select resistance-associated bacteria that could confer resistance to *C. difficile* infection in a secondary bile acid dependent manner.^47^ Stein et al. established a microbiome-immune system mathematical model by incorporating data from regulatory T-cells and microbiota composition to predict ecologically stable defined microbial consortia in promoting T-reg activation. While the small to moderate number of bacterial isolates within a synthetic community may not encapsulate the natural diversity found in the gut, as the examples described above have demonstrated, it can provide a representative and functional substitute. In addition, various combination options of different microbial strains can potentially allow for generation of personalized synthetic gut microbiotas for precision medicine in the future.

Another challenges is that different gut microbial species have different nutritional and physiological growth requirements, which makes the generation, culture, co-culture, and preservation of these communities very difficult and exact reproducibility very challenging.^48^ While more efforts are still needed in evaluating their representability and efficacy compared to complete fecal microbiota, synthetic microbial communities represent a promising alternative for FMT due to relatively easily controlled composition and the potential for standardization over FMT. Mabwi et al. have conducted a thorough review exclusively on current advances and challenges of synthetic microbial communities should there be further interest in this topic.^48^

### 2.3 Next-generation probiotics

Classical probiotics are usually strains of *Lactobacillus* and *Bifidobacterium*. Along with the characterization of human gut microbiota, new types or next-generation probiotics (functional bacteria with beneficial and therapeutic properties) are being discovered, developed, and used for disease treatment.^49,50^ There are thousands of clinical trials registered with *ClinicalTrials.gov* which are investigating the therapeutic effects of various individual probiotic bacterium and probiotic combination treatments for a wide range of diseases and conditions. *Akkermansia muciniphila* is one of the most promising next-generation probiotics as it is the most frequently reported gut microbial species that have beneficial effects on the host. *A. muciniphila* is abundant in the gut (0.5–5% of the total bacteria) and known as a mucin-utilizing bacterium.^51^ Numerous studies have shown that *A. muciniphila* was inversely associated with a variety of diseases, including obesity, diabetes, cardiovascular diseases, and low-grade inflammation.^52^ Both live bacterium and pasteurized *A. muciniphila* showed beneficial effects on body weight, glucose tolerance, and insulin resistance in animal models.^53^ Species from the genus *Blautia* were also commonly associated with anti-inflammatory effects and thereby can be a promising therapeutics as well.^54^ Sen *et al*. reported that in an ASD mouse model, oral administration of *B. stercortis* MRx0006 attenuated social deficits and anxiety-like behavior, indicating that this strain can be an efficacious treatment option for the management of disorders associated with ASD.^55^

Although there are many possible health and therapeutic benefits, there are also some clinical scenarios where probiotics can cause unwanted side effects or worse outcomes that merit consideration as well. For example, despite the promise of *A. muciniphila* as a therapeutic against multiple diseases as discussed above, there are also some clinical scenarios such as in graft versus host disease where increased abundance of *A. muciniphila* is associated with worse patient outcomes as a result of its mucus degrading abilities, causing loss of the colonic mucus layer and increased intestinal inflammation.^56^ In addition, probiotics usually can’t be used as a blanket replacement or substitute for other microbiome-directed therapies, as, for example, in post-antibiotic gut microbiome recovery, the use of probiotics delayed reconstitution while autologous FMT provides health benefits.^57^

Probiotics are generally a purer/cleaner form of bacterial-delivery based therapy, with known composition and manufacturing contents, and ability for individualization. However, there are currently extremely limited therapeutic regulations of probiotics as they are considered nutritional supplements rather than drugs. Currently, probiotic therapeutics are usually regulated as LBPs or advanced therapeutic products (ATPs), which require highly tailored regulatory approaches to ensure protection of the safety and health of people. There are still questions when it comes to shelf-life for probiotics and the impacts of probiotic viability as viable and non-viable microbes can actually provide varying beneficial effects.

### 2.4 Engineered bacterial strains

Along with the development of synthetic biology, genetically engineered microorganisms to specifically address disease mechanisms is also emerging.^58^ Isabella et al. engineered a probiotic strain *E. coli* Nissle to express genes encoding enzymes that specifically metabolize phenylalanine (Phe) and found that administration of this engineered probiotic strain efficiently reduced the blood Phe level in both mouse and primate models of phenylketonuria (PKU).^59,60^ A first-in-human clinical trial also supported the potential use of this engineered bacterium for the treatment of rare diseases like PKU.^59^ This synthetic biology technique has been applied to generate bacterial strains that can target the stimulator of interferon gene (STING) pathway for cancer therapy as well.^61^ Ho et al. engineered commensal *E. coli* to provide selective affinity to cancer cells and secrete myrosinase for converting vegetable derived glucosinolate into anti-cancer compounds, which showed desired anti-cancer activity against colorectal cancer in both murine model and *in vitro* cell lines.^62^

Antimicrobial resistance (AMR) is an emerging silent pandemic that threatens the healthcare system.^63,64^ Recent studies have shed light on the use of engineered probiotics for addressing the AMR accumulation and spread in the microbiome. For example, Cubillos-Ruiz et al. designed an approach to engineer a β-lactamase-expressing probiotic *Lactococcus lactis* strain to degrade broad-spectrum antibiotics β-lactams.^65^ Oral supplementation of this probiotic in mice treated with parenteral ampicillin obviously minimized the gut dysbiosis and prevented the accumulation of AMR genes in the microbiome. Koh et al. demonstrated that intestinal bile salt metabolism was disrupted by antibiotic treatments, which may contribute to the development of recurrent CDI.^66^ Supplementation of an *E. coli* Nissle strain engineered with a genetic circuit to control intestinal bile salt metabolism effectively inhibited the growth of *C. difficile* and colitis phenotypes in mice, indicating a promising antimicrobial strategy without disrupting the intestinal symbiosis.

### 2.5 Bacteriophages

Bacteriophages are viruses that target bacteria with high resolution of specificity and are considered as promising treatments against antibiotic-resistant bacterial infections.^67^ When applied for the manipulation of the microbiome, bacteriophage has the advantage of high specificity, genomic plasticity, and multiplication rates.^68^ Duan et al. reported that cytolysin-positive (cytolytic) *Enterococcus faecalis* is associated with hepatocyte death and liver injury, leading to the development of alcoholic liver disease in both human and animal models.^69^ They further demonstrated that *E. faecalis* specific bacteriophages that were isolated from sewage water efficiently decreased cytolysin in the liver and abolished ethanol-induced liver disease in humanized mice.^69^ More recently, Federici et al. reported a strategy to develop a five-phage combination therapy that can precisely suppress IBD-associated gut pathogen *Klebsiella pneumoniae* and consequently alleviate inflammation in IBD animal models.^70^ A first-in-human phase 1 clinical trial demonstrated that this five-phage therapy is safe, viable and can be accumulated in the lower gut in healthy adults, representing a promising therapeutic candidate for IBD treatment.^70^

### 2.6 Postbiotics and antimicrobial peptides

Postbiotics are functional bioactive compounds or by-products generated during fermentation, they include but not limited to vitamins, cell wall components, proteins, peptides, short-chain fatty acids (SCFAs), exopolysaccharides (EPS), and extracellular vesicles (EVs) which may be used to promote human health.^71,72^ The intestinal lumen is rich in metabolites/proteins that are derived from both the host and microbes, and are important in maintaining the healthy intestinal microenvironment. Alterations of this chemical pool can be a contributing factor for the development of diseases.

Postbiotics can have direct or indirect effects to benefit the host, microbiome and their interactions. EPS derived from a variety of sources, ranging from different bacterial species to fungi, have been found to have bifidogenic effects.^73,74^ This means EPS can modulate the gut microbiome composition by specifically enhancing the growth of bifidobacteria, which are a probiotic group of bacteria that normally reside in human gut to confer health benefits to their host. SCFAs are another major type of postbiotics, which can either be used in microbial cross-feeding, or directly impact colonocytes and modulate cellular activity within the gut/colon.^71^ Wegh *et al*. have conducted a thorough review exclusively on the mechanisms and applications of known postbiotics should there be further interest in this topic.^71^

Antimicrobial peptides/proteins (AMPs) are a class of biomolecules that are widely present in different organisms, including human, bacteria and fungi.^75^ While most of the currently known AMPs are host derived defense proteins/peptides, other organisms such as microbiota are also important producers for AMPs.^76^ AMPs possess a wide spectrum of antimicrobial activities against bacteria, virus, fungi and parasites, as well as have immune-modulating activities.^77^ AMPs function through multiple mechanisms of action; there are multiple proposed models by which AMPs ultimately disrupt the integrity of bacterial membranes, and it’s also been found that they can translocate into the target bacteria and inhibit a variety of critical cell functions such as DNA/RNA and protein synthesis.^77^ More recently, AMPs have been demonstrated to mediate host-microbe interactions in the gut for maintaining intestinal homeostasis.^78^ These unique features make AMPs a promising therapeutic for both fighting against pathogens and regulating host immune homeostasis. Currently, over 30 AMPs are being evaluated in clinical trials for treating diseases that are caused by pathogens, such as *C. difficile*, methicillin-resistant *S. aureus* (MRSA), and *Candida*.

## 3. Multi-omics for characterizing the microbiota-type therapeutics

Given the high complexity of the gut microbiota, a pure culture-independent -omics approach is needed to comprehensively characterize microbiota or microbiota-type therapeutics.^9^ Along with the development of next-generation sequencing (NGS) and high-resolution mass spectrometry (MS) methods, multiple meta-omics approaches have been developed to efficiently profile the taxonomic compositions (meta-taxonomics), genes (metagenomics), transcripts (metatranscriptomics), proteins (metaproteomics), or metabolites (meta-metabolomics) in complex microbial community.^9^ It is the rapid development and wide application of meta-omics that drove the explosion of our understanding on the roles of microbiome in human health and diseases in the past decade. The readers are encouraged to refer to previous review articles that extensively summarized the methodology and application of each meta-omics approach.^9,79–81^ Here we focus on the comparison of different meta-omics in considering their translational potentials, in particular the CMC (Chemistry, Manufacturing, and Controls) and clinical use of microbiota-directed therapeutics (Table 2).

**Table 2.** Overview of meta-omics methods for the characterization of the gut microbiota and microbiota-directed biotherapeutics.

-Omics	Input	Sample multiplexing	Depth	Readouts	Limitations	Advantages
Metataxonomics	16s rDNA 18S rDNA ITS	High	High	Taxonomy (genus or higher level)	Low resolution Bias due to primers selection No functional information	Low cost Well established bioinformatics tools Targeted detection of rare disease-associated microbial species
Metagenomics	Genomic DNA	Low	High	Taxonomy (species/strain level) Gene abundance Function/pathway potential	High sequencing depth is needed Can’t discern expressed and not expressed functions	High-resolution taxonomics (strain level) Enable genome-level analysis, such as genome-scale metabolic reconstruction Detection of specific genes and pathways
Metatranscriptomics	mRNA, can extend to other RNAs (cDNA)	Low	High	Taxonomy (species/strain level) Transcript abundance Active function/pathway	High sequencing depth is needed RNA instability Complicated sample preparation protocol Complicated bioinformatics workflow	High-resolution taxonomics (strain level) Detection of functional activity of microbiomes
Metaproteomics	Proteins/peptides	Low (up to 18-plex)	Low	Taxonomy (species/strain level) Protein abundance PTMs Biomass Host proteins	Low measurement depth Complicated bioinformatics workflow High dynamic range of protein abundances	Detection of functional activity Detections of protein isoforms (e.g., PTMs) and protein-protein interactions Detection of proteins derived from the host (as host biomarkers) Enable absolute biomass estimates
Metabolomics	Metabolites, including lipids	Low or None	Low	Metabolite concentrations (host and microbial origin)	Mixture of host and microbe metabolites Insufficient identification of metabolites Low measurement depth and usually need different data acquisition modes	Direct measurement of key metabolites Easy data interpretation

Meta-taxonomics examines phylogenetic marker sequences (e.g., 16S rRNA gene, 18S rRNA gene, and internal transcribed spacer (ITS) gene) using amplicon sequencing and is ideal to profile the structural composition of microbiota. 16S rRNA gene sequencing is the most widely used meta-taxonomic approach in studying microbiome and has the advantage of high multiplexing capability, low cost, and well established bioinformatic pipelines (such as QIIME2, DADA2 and UPARSE).^82–84^ Therefore, 16S rRNA gene sequencing is most likely to be widely applied for standardized characterization of individual’s microbiotas and microbiota-directed therapeutics. Another important advantage of amplicon sequencing (or taxonomics) is that it can better detect rare or low abundant taxa than other -omics approaches because it is a targeted method, which can be very important when looking at very uneven and heterogeneous fecal microbiota populations. However, meta-taxonomic approach is limited to the composition characterization usually with low resolution (genus or higher level) and is unable to provide functionality information of the microbiome, although some tools such as PICRUSt can predict functions from taxonomic composition data.^85^

Whole genome shotgun metagenomic sequencing is increasingly applied for the study of microbiomes with high phylogenetic resolution (down to strain level) and provides information on the functional potential (i.e., encoded genes) of the microbiomes.^86^ Similarly, metatranscriptomics uses shotgun sequencing to examine the transcribed mRNA sequences in microbiomes, which provides more functional information on the microbiome’s functionality.^87^ A few comprehensive bioinformatic pipelines/tools have been developed and commonly used to generate either taxonomic (such as MetaPhlAn2 and Kraken2^88,89^) or functional profiles (such as HUMAnN2 and MOCAT2^90,91^) of microbiome from the complex shotgun sequencing data. While multiplexing is possible, more sequencing depth is needed for metagenomics and metatranscriptomics to achieve enough coverage of the low abundant microbial genomes. This limits their multiplexing capacity, increases the cost, and thereby hampers their wide application in large scale or high throughput applications. Nevertheless, along with the development of more advanced sequencing platforms with higher speed and lower cost, NGS-based meta-omics methods will be promising tools for biomarker discovery, diagnosis, and the entire lifespan of microbiota-directed drug development. The latter includes the quality assessment of microbiota-type therapeutics and the development of potency assay, which is a regulatory requirement for lot release of all approved therapeutic products.

While NGS provides valuable information on the encoded or transcribed genes in the microbiome, it is still unknown whether these genes or transcripts will result in protein expression and metabolite synthesis. The latter two are the direct mediators of the interactions between microbiota and the host. Therefore, accumulating studies are examining the proteins, namely metaproteomics, and metabolites, namely meta-metabolomics, to better characterize the microbiota functionality.^9^ Most current metaproteomics and meta-metabolomics studies were carried out based on high-resolution MS coupled with high- or ultra- performance chromatography.^92^ Briefly, proteins or metabolites can be extracted from samples using mechanical and/or chemical extraction methods; the extracted proteins are then digested into peptides and metabolites are derived or directly injected for chromatography separation and MS measurements.^92^ Unlike NGS-based – omic approaches that require more sophisticated molecular techniques, such as polymerase chain reaction (PCR) and sequencing library construction, MS-based meta-omic approaches usually adopt simpler and streamlined sample preparation workflows, such as the single-pot, solid-phase-enhanced sample-preparation (SP3) method^93^ (Table 2). While it’s challenging to perform multiplexed sample analysis for metabolites, metaproteomic analyses can be multiplexed using isobaric tandem mass tags (TMT), which allows up to 18-plex sample measurement and the labeling workflow can be streamlined for high throughput applications.^94,95^ In addition, metaproteomics has unique capabilities to examine diverse protein isoforms, including post-translational modifications (PTMs), which are critical for protein activity and thereby microbiota functionality.^96–99^ Bioinformatic tools for processing metaproteomic and meta-metabolomic data were developed and optimized only very recently. For example, MetaLab and MetaProteomeAnalyzer are commonly used metaproteomic workflows.^100,101^ While there is no bioinformatic tool specifically designed for meta-metabolomics, MetaboAnalyst and XCMS are commonly used for metabolomic analysis of samples, including microbiome samples.^102,103^ Along with the development of more advanced MS platforms (such as instruments that enable high-resolution single-cell measurements) and more dedicated and easy accessible bioinformatic tools, MS-based -omics approaches are expected to be widely applied in microbiome characterization as well.

Given the advantages and disadvantages of each meta-omic approach, integrative multi-omics approaches have also been applied to the study of microbiome in various diseases, such as type 1 diabetes and inflammatory bowel diseases.^104,105^ In addition to the significant increase of time and cost needed for multi-omic measurement, the development of bioinformatic and statistical tools/pipelines that truly integrate multiple -omic datasets to efficiently extract meaningful information is even more challenging and more efforts are warranted. Another limitation for most current meta-omics approaches is that they only examine relative abundance of microbes or molecules without measuring the overall microbial load. The latter itself has been demonstrated to be associated with diseases^106^ and could be a key attribute for evaluating microbiota-type therapeutics. By integrating 16S rRNA gene sequencing and flow cytometric enumeration of microbial cells, Vandeputte et al. developed a quantitative microbiome profiling workflow that demonstrated important role of microbial load in influencing the enterotype and microbiota alterations in Crohn’s disease.^106^ Metaproteomics measures the abundances of proteins, which could be a measure of biomass of microbial populations or community.^107^ By using an equal volume based sample preparation workflow, Li et al. demonstrated that metaproteomics enabled absolute biomass assessment and revealed an inhibitory effect of antibiotics and several non-antibiotic drugs on the growth of microbiome.^108^

## 4. Functional assays for evaluating microbiota-directed therapeutics

To provide efficient quality assessment of microbiota-type therapeutics, assays or experimental models that enable evaluation of microbial viability, bioactivity and the interactions with the intestinal cells are needed. Animal models have been widely used in microbiome research, and transplantation into germ-free mice was used to evaluate whether the microbiota is active or alive.^109^ However, these animal experiments are time consuming and expensive for large-scale drug screening and routine quality assessment. Recent developments of *ex vivo* microbiota culture mediums and methods have enabled efficient *in vitro* maintenance of microbiota structure and functionality. These assays enable economic and high throughput studies of drug-microbiota interactions and are promising assays for assessing the microbiota-directed therapeutics. *In vitro* intestinal cellular models were widely applied for evaluating the drug permeability or their effects on barrier functions,^110,111^ which can be adapted for the evaluation of host modulating effects of microbiota therapeutics. In addition, assays that enable direct assessment of the immune-modulating effects of microbiota, such as T cell repertoire assay,^112^ is also an important component of the toolbox. Some examples of and current developments in functional assays which can be used for evaluating microbiota-directed therapeutics are discussed below.

### 4.1 Ex vivo microbiome assay

To evaluate drug-microbiome interactions, conventionally, individual gut bacterium is isolated and cultured *in vitro* with drugs. For example, Maier et al. performed a high throughput screening of > 1000 FDA approved drugs against 40 human gut microbial strains and showed that 24% of the non-antibiotic drugs could impact specific gut bacterial species, suggesting extensive drug-microbiome interactions.^16^ Zimmermann et al. also reported that around two-thirds of their selected 271 oral drugs were metabolized by at least one of the 76 cultured human gut bacterial strains.^18^ While individual bacterium culturing provided a rapid and easy assay for drug-microbe interaction study, it did not represent the whole microbial community. Herberth and von Bergen et al. developed a simplified human intestinal microbiota (SIHUMIx), consisting of eight common human intestinal bacteria, in a continuous flow bioreactor,^113,114^ which maintains a stable microbial community and may act as a platform for evaluating the microbiota-targeted therapeutics. This provided a useful, reproducible, and easy to manipulate system to study the effects of various factors on a microbial community model. However, synthetic microbial communities still do not fully represent the whole microbiota and the continuous flow setup is not compatible with high throughput assay development.

Researchers have made great efforts in culturing the entire microbiota in the past few decades, such as the use of artificial gut system to mimicking intestinal and colonic physical conditions. Artificial gut systems are typically variations of multi-compartment reactors which allow the long-term maintenance of inoculated microbiota through the control of many environmental conditions across multiple compartments which reflect various conditions along the intestinal tract. These conditions include but are not limited to the control of nutritional input, pH, time spent in as well as composition of gastric acids, temperature, and the incorporation of peristaltic pumps to provide an accurate recreation of the physical conditions present *in vivo*. An example of a classical artificial gut system is the simulation of the human intestinal microbial ecosystem reactor, or SHIME reactor, which featured a 5-step multi-chamber reactor.^115^ The SHIME model included compartments, which simulate the stomach, small intestine, ascending, transverse, and descending colon representing both the upper and lower digestive tract.^115^ Another example is *T*NO computer-controlled, dynamic *in vitro* gastro-*I*ntestinal *M*odel of the colon (TIM) which has a multi-compartmental design to accurately recreate the dynamic conditions of the gut and colon respectively, and has accurately predict clinical trial outcomes.^116,117^ The TIMs systems both utilize a flexible membrane to allow better movement of the components, with TIM-1 recreating the stomach, duodenum, jejunum, and ileum as the gut model, and TIM-2 modified to recreate the large intestine^116,117^. Through the alteration of nutritional input, microbiota input, or any of the many dynamic physical parameters such as peristaltic movements, fluctuating pH, or timing within components – the system can be adapted to simulate a wide array of target conditions such as species, age, nutritional status, and health status.^116,117^ The inTESTine system takes artificial gut systems one step further and incorporates and uses porcine intestinal tissue and contains mucus layer which can greatly affect nutrient and drug absorption, bacterial colonization, and immune responses.^118^ These and other artificial gut *ex vivo* systems can accurately predict clinical outcomes and are incredibly useful in the study of gut microbiota. They are important tools in studying microbiome and gastrointestinal systems as they can effectively replace many *in vivo* experiments and allow the control of a large quantity of dynamic conditions. The complexity of these systems is an advantage when recreating various gut-systems under different contexts or when looking at the contribution of different factors, but can also be a disadvantage for ease of establishment and use.

*Ex vivo* batch culturing of entire human gut microbiota provides another promising way which is compatible with high throughput applications. One challenge is to maintain the composition and functionality of the gut microbiota in *in vitro* batch conditions. Recent studies have identified key nutrients for microbiota growth and consequently optimized the microbiota culturing mediums that can maintain both microbiota functionality and composition.^119–121^ Li et al. developed RapidAIM,^108^ a 96-well plate-based microbiome assay enabling cost-effective and high-throughput microbiota-targeted drug screening, which has also been used for evaluating various substrates, including FDA-approved drugs, resistant starch and natural compounds.^122–124^ *Ex vivo* microbiome culturing system has also been applied for the rapid screening and detailed characterization of microbiome-derived drug metabolism when combined with targeted metabolite analysis.^18,125^ The batch culturing platform is cost-efficient, easy-to-implement, and can be readily multiplexed for different purposes, including drug screening, study of drug-microbiota interactions, and the evaluation of fresh and banked microbiota or FMT viability/cultivability.^62^ For example, we have previously applied the RapidAIM assay and metaproteomics for evaluating live microbiota biobanking, which demonstrated that up to one year of freezing in a deoxygenated glycerol buffer had minimal detrimental influences on the cultivability of fecal microbiota.^62^ We also showed that delayed sample processing was possible for 48 hours if the microbiota were kept on ice in a deoxygenated buffer, but not on dry ice.^62^

### 4.2 *In vitro* intestinal cellular models

The complement to the *ex vivo* microbiome assay are the assays based on *in vitro* intestinal cellular models, such as immortalized cell lines, cell line co-cultures, 3D intestinal cell culture, microfluidics-based cell culture and organoids (Table 3). *In vitro* intestinal epithelium models are well suited to evaluate how various therapeutics, bacterial secretions and surface markers, or microbiota themselves affect the host epithelial functions. The most widely used *in vitro* intestinal cellular model is the Caco-2 immortalized cell line. It is a colorectal adenocarcinoma line that grows as a monolayer. Upon reaching confluence, Caco-2 cells undergo spontaneous enterocytic differentiation – adopting morphological and functional similarities to a human small intestine.^126^ There is a plethora of published differentiation methods for Caco-2 cells, ranging in the use media additives such as butyrate, seeding density, length of differentiation (ranging from 7 to 21 days), static versus microfluidic perfusion culture, use of transwell inserts for polarization of cells, as well as the use of scaffolds to recreate various structures during *in vitro* culture.^133,139,140^ While differentiated Caco-2 cells alone provide an efficient and easy model to test host responses, it misses many factors that are present *in vivo*, such as the intestinal mucus layer and the interactions between various cell types, leading to great efforts to develop co-culture models. For example, Antunes et al. developed a triple co-culture *in vitro* model including Caco-2 as intestinal epithelial cells, HT29-MTX as mucus-producing goblet cells, and Raji B cells that induced a luminal-sampling M-cell phenotype, an important element for immune response, leading to a complex, polarized model which more closely represents the behavior of a human gut.^127^ To increase the model morphological accuracy, there has also been many studies developing 3D culture of intestinal epithelial cells using various scaffolds.^128–130^

**Table 3.** Advantages and limitations of different *in vitro* intestinal models for evaluating microbiota-directed therapeutics.

*In Vitro* Cellular Model	Description	Example(s) and References	Advantages	Limitations
Immortalized cell lines	Cell cultures that have been modified to divide indefinitely	Caco-2 cells (undergoes spontaneous enterocytic differentiation to adopt morphological and functional similarities to a human intestine) ^126^	Easy model to work with and maintain Inexpensive and widely available for use Well-characterized for many cell types High throughput capacity No need for overly complex or specialized equipment	Do not fully mimic the behavior and function of primary cells isolated directly from the issue of interest Misses many factors that are present *in vivo* (ex: intestinal mucus layer, interactions between various cell types, immune functions) Only host cells and host response is observed Does not model the complex interactions between different cell types found *in vivo*
Co-culture of cell lines	Culture of a combination of cell lines in the same culture dish or system	Co-culture of Caco-2 cells, HT29-MTX, and Raji B cells, adding mucus production and immune response elements to generate a complex morphological and functional cell culture model of the human intestine^127^	Allows for the study of cell-cell interactions Efficient and straight forward model to test host responses More closely mimics the *in vivo* microenvironment (can incorporate mucus layer, immune functions) than single immortalized cell culture leading to more biologically relevant results No need for overly complex or specialized equipment High throughput capacity	More technically challenging and time-consuming than using immortalized cell lines Only host cells and host response is observed Does not fully reproduce the complex microenvironment found *in vivo*
3D cell culture	Culture of cells in a 3D environment, can be achieved using various scaffolds or gels	Caco-2 cultured with electrospun scaffolds, hydrogels, 3D-printed scaffolds, or subepithelial-like tissue constructs containing fibroblasts ^128–130^	More closely mimics the *in vivo* microenvironment than standard monolayer culture Higher morphological and physiological model accuracy Improved epithelial cell polarization Includes effects of extracellular matrix molecules More representative paracellular permeability	Low throughput compared to monolayer cell culture More technically challenging and more costly than monolayer cell culture Only host cells and host response is observed Does not fully reproduce the complex microenvironment found *in vivo*
Microfluidics-based co-culture systems	Co-culture of differentiated human epithelial cells with facultative anaerobic or anaerobic bacterium under both aerobic or anaerobic conditions	HuMiX,^131^ MIMICS^132^	Provides the ability to study host-microbe interactions Allows for precise control of the microenvironment, including the composition, flow of media and oxygen levels Scalable and automatable for high-throughput	Can be costly to set up and maintain May not be suitable to all cell or bacteria types More technically challenging to set-up than regular monolayer cell culture Does not fully reproduce the complex interactions between different cell types found *in vivo*
3D cell culture in microfluidics co-culture system	Morphological features of an intestine, such as villi and crypt structures, which can be colonized by living bacteria and allows the study of host-microbiome interactions in an immunocompetent environment from a readily accessible immortalized cell line	Organ-on-chip^133^	Higher morphological and physiological model accuracy Provides the ability to study host-microbe interactions Allows for precise control of the microenvironment, including the composition, flow of media and oxygen levels	Can be costly to set up and maintain May not be suitable to all cell or bacteria types More technically challenging to set-up than regular monolayer cell culture Does not fully reproduce the complex interactions between different cell types found *in vivo*
Organoids	Stem cell derived, self-organizing, 3D tissue-like structures, which closely mimic a tissue of interest	iPSC- or patient-derived intestinal organoids^134–137^, iHACS (Intestinal Hemi-Anaerobic Co-culture System)^138^	More closely mimic the microenvironment and cell-cell interactions found *in vivo* Higher morphological and physiological model accuracy Can be used to study the development and function of organs over time, as well as to model diseases and test potential therapies Provides the ability to study host-microbe interactions	Time-consuming and costly to culture and expand organoids More technically challenging to set-up experiment and the need of micro-injection to study host-microbe interactions Difficult to scale up for use in high-throughput studies

In a shift from solely looking at the host response to host-microbiome interactions, many groups are developing approaches to culture bacterium and human epithelium together to better investigate those interactions. Shah et al. developed a modular microfluidics-based human microbial co-culture model (HuMiX) which permitted the co-culture of differentiated human epithelial cells with facultative anaerobic or anaerobic bacterium under both aerobic or anaerobic conditions, providing the ability to study human-bacterial interactions of the gut in more representative culture model.^131^ Song et al. also developed a Mimetic Intestinal Host – Microbe Interaction Coculture System (MIMICS) which allowed them to culture probiotic candidate *A. muciniphila* with Caco-2 cells and investigate the host-microbiome interactions of a live probiotic on intestinal epithelial cells with meta-omics approaches.^132^ Maurer et al. developed a 3D cell culturing method to produce an organ-on-chip model with morphological features of an intestine, such as villi and crypt structures, which can be colonized by living bacteria and allows the study of host-microbiome interactions in an immunocompetent environment from a readily accessible immortalized cell line.^133^ Zhang et al. developed a cell culture method that utilizes fluidics to generate a steep oxygen gradient allowing the extended co-culture of aerobic human epithelial cells with a strictly anaerobic bacterium *Faecalibacterium prausnitzii*.^141^ Given the importance and beneficial effects of bacterium such as *F. prausnitzii* on inflammatory and immune responses within the gut, having the ability to study those interactions as well as pathogens or biotherapeutics in a context that takes both the host and microbiome into account simultaneously are important to provide clinically applicable insights.

A different approach to modeling the intestine *in vitro* started with the discovery and use of Lgr5+ stem cells, which are located in the crypts of the small intestine responsible for the constant self-renewal of intestinal tissue in humans, to produce organoids.^134,135^ Organoids are stem cell–derived, self-organizing, 3D tissue-like structures, which closely mimic a tissue of interest.^135–137^ Mead et al. have recently published the use of *in vitro* intestinal organoids in a high-throughput screening study to search for tissue-modifying agents, demonstrating that the use of stem cell organoids can be scalable.^136^ Mithal et al. have shown that human intestinal organoids can also be formed using human induced pluripotent stem cells.^137^ This emerging patient-specific tool, coupled with gene editing techniques, allows the isogenic comparison of normal and disease intestinal organoids and supports large-scale drug screening applications or personalized medicine. Akin to the growing interest of co-culturing of bacterium with immortalized cell line based intestinal *in vitro* models, co-culture of intestinal organoids and probiotics, symbionts, and pathogens to study the host-microbiome interactions is an emerging field of interest.^142–144^ Sasaki et al. developed a method of co-culturing intestinal organoids with anaerobic bacteria which have different oxygen demands, by dissociating 3D cultured organoids and seeding them into a 2-chamber culture system called the Intestinal Hemi-Anaerobic Co-culture System (iHACS) allowing for simultaneous hypoxic and normoxic co-culture of human and bacterial cells.^138^

### 4.3 Assays for profiling immune-microbiota interactions

Intestinal epithelium models can be used to evaluate how the microbiota impacts the host epithelial functions; however the understanding on how the microbiota or their components interact with immune systems, in particular the mucosal immunity, is critical for assessing microbiota-type therapeutics. Previous studies on immune-microbiota interactions were usually performed using gnotobiotic animal models or freshly isolated primary immune cells. However, these approaches are expensive, time-consuming, low throughput and difficult to implement for drug evaluations. T cell response provides the widest spectrum of antigen recognition in the gut mucosal immune system and may participate in enabling B cell produced immunoglobulin specificity.^145^ By using single cell RNA and T cell receptor (TCR) sequencing, Nagashima et al. identified potential microbiome-specific T cell clonotypes and successfully constructed 92 T cell hybridomas with each expressing a single microbiome-specific TCR. These T cell hybridomas were then used for high throughput mapping of T cell repertoire to individual bacterial strain in a microbial community.^112^ Compared to primary T cells and T cell lines, T cell hybridomas have the advantages of easy and rapid growth in cell culture, relatively high uniformity and stability,^146^ and therefore are well suited for assays to evaluate microbiota-immune interactions. Instead of using IL-2 production as a measurement of T cell stimulation, Mann et al. developed an 8-plex multiplexed T cell hybridoma assay based on the expression and measurement of different fluorochromes for simultaneously screening the stimulations of multiple T cell hybridomas,^147^ representing a promising assay system for evaluating immune-modulating activity of microbiota or the derived therapeutics.

Immunopeptidomics is an approach for directly profiling the repertoire of major histocompatibility complex (MHC)-presented peptide antigens using MS, which has been proven to benefit vaccine development for both cancer and infections.^148,149^ Briefly, MHC-bound peptides are purified from patient samples or cultured cells that were infected with pathogens; the eluted peptides are then subjected to MS for peptide identification. In recent years, the MS sensitivity for peptide analysis has been dramatically increased, which enables the identification of thousands of unique and low abundant peptide antigens in a single experiment. The profiling of MHC peptide repertoire could therefore be a promising approach to evaluate immune-modulating activity of drugs, including microbiota-directed therapeutics. Stopfer et al. developed a quantitative immunopeptidomics approach by spike-in of heavy isotope-coded peptide MHCs and demonstrated alterations of immunopeptidome profiles in response to CDK4/6 inhibition in melanoma cell lines.^150^ To the best of our knowledge, there is still a lack of immunopeptidomic study on microbiota due to its extremely high complexity and bioinformatic challenges. However, with the technical advances, profiling and quantifying meta-immunopeptidome will be possible, which may provide an alternative method for assessing the immune-modulating activity of microbiota-type therapeutics.

In a time where replacement, reduction, and refinement of animal studies is an ethical and scientific priority, having such complex and representative *in vitro* or *ex vivo* models is very valuable in understanding the effects of therapeutics on both host and microbiome, and can deepen our understanding of microbiome-based therapeutics.

## 5. Clinical and regulatory challenges for microbiota-directed biotherapeutics

Along with more microbiota-directed biotherapeutics being developed and applied for clinical use, the current healthcare delivery system is likely to be markedly impacted.^151^ From the clinical point of view, as the microbiome is associated with various types of diseases and can affect drug efficacy, the knowledge on microbiome science is then critical for physicians to better prescribe medications and provide medical or dietary advice for patient care. It is possible that measurements of gut microbiota become incorporated as part of physicochemical examination for guiding diagnosis and treatment decision. All these require that our frontline healthcare workers are equipped with scientifically sound knowledge on microbiomes. However, the field of microbiome science itself is still in its infancy. Our understanding on the roles of microbiome is rapidly evolving, while many key questions remain unanswered. For example, it’s unclear whether we can define what a healthy microbiota means, and whether the microbiota changes are a cause or consequence for many diseases.^8^ Nevertheless, incorporating microbiome science for medical education can be a necessary step in preparing for the microbiome-directed medicine era.

As one of the most successful examples, FMT is now a standard-of-care for CDI patients that are recurrent or resistant to antibiotic treatment.^22,23^ However, only limited numbers of hospitals are capable of implementing FMT, which greatly limits the patient accessibility. Usually, FMT is implemented by colonoscopy or enema with fresh or frozen fecal materials from self-stool banks or donors that are known to the medical team or recipients. However, sourcing a proper donor for FMT is challenging and expensive given the need for extensive screening of the donors as well as the fecal materials themselves.^152,153^ Stool banks, such as Openbiome, is an alternative way to provide easy-to-access and safe material for FMT.^154^ However, further regulatory, ethnic and financial considerations are still needed for sourcing FMT from stool banks. Recently Rebyota®, a microbiome therapy, has received FDA approval as a treatment for recurrent CDI,^155^ which is a great step forward for microbiome-based therapies in the clinic. There is still an urgent need to develop easy-to-access dosage forms of FMT, such as oral capsules, and other LBPs as proxies of FMT.

**Figure 1.**
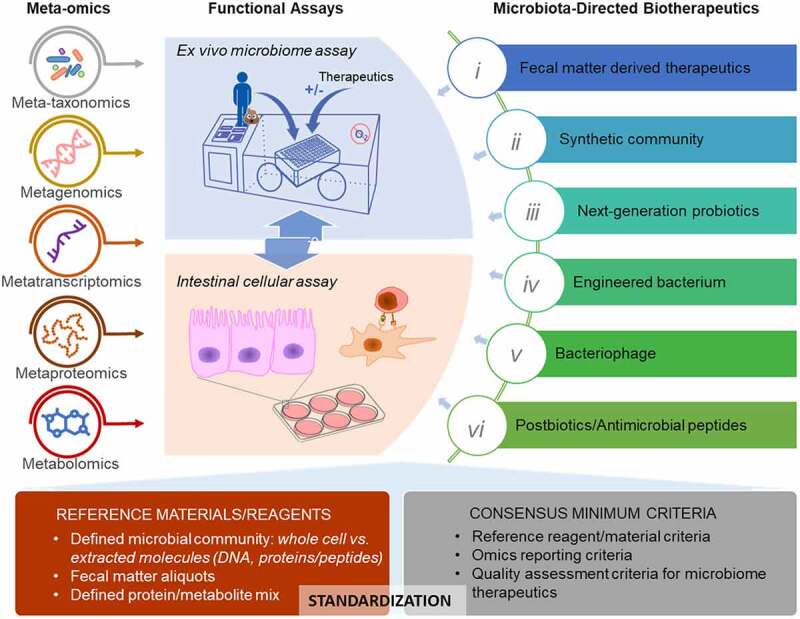
Characterization of the microbiome and microbiome-directed biotherapeutics using multi-omics and *in vitro* functional assays.

The emerging microbiome therapeutics also pose a challenge to the health authorities for efficient quality assessment and regulations. The current platforms and approaches for regulating therapeutics are mainly designed for chemical xenobiotics or conventional biologics, such as mAb and vaccines, which target the individual pathogens or human cells themselves. There is no widely accepted approach for the regulation of advance therapeutics, such as FMT. Different health authorities regulate FMT in different ways with some considering FMT as a biologic, such as Health Canada and FDA, while others regulate it as a tissue or drug.^22,23,156^ Great efforts have been made in extensive donor screening for FMT, such as the timely updated screening criteria to include SARS-CoV2 and monkeypox virus. However, limited or insufficient efforts have been made to characterize the fecal matter itself. The classical approach of microorganism characterization using agar plate culturing and colony visualization lacks resolution to enable safe and efficient quality assessment given that a healthy microbiota could have>200 different microbial species with distinct physiological properties.^2^ Other non-bacterial components, such as fungi, archaea, and bacteriophages, have also been well demonstrated to have critical metabolic/pathogenic functions.^4–7^ Currently, there is still a lack of consensus on the definition of a healthy microbiota and the minimum components that enable efficient beneficial microbiome functionality are unknown. Therefore, the assessment of microbiome therapy requires advanced techniques, such as multi-omics, which can efficiently characterize the species and biochemical composition of the product. It also requires unique functional assays for the evaluation of microbiota viability and functional activity (Figure 1).

Recent development of -omics approaches, including metagenomics and metaproteomics, has revolutionized the way to characterize the microbiome (details in section 3).^9^ Although multi-omics is valuable and informative, the application of multi-omics is still largely limited for research and potential biomarker discovery. It is known that every step of multi-omics analysis, including molecule extractions, sample preprocessing, instrumental measurement, and downstream bioinformatic analysis, can result in bias to the final results.^157,158^ While there is a risk of reduced innovation, standardization of the multi-omics sample preparation, data generation, analyses, and interpretation is needed for application in clinical and regulatory practices. Generation of reference materials for microbiome analyses is one important way to benchmarking methodologies for consistency and efficiency (Figure 1). Different types of reference materials, including extracted total DNA mixture or lyophilized microbial cells of well-defined microbial community,^159,160^ have been developed or commercially available (e.g., Zymo ZymoBIOMICS® and ATCC NGS standards). Aliquots of raw fecal matter have also been used in some benchmarking investigations.^161,162^ In addition to the reference reagents, general guidelines and consensus minimum criteria for data reporting are also critical (Figure 1). Amos et al. developed a four-measure reporting framework, including sensitivity, false positive relative abundance (FPRA), diversity, and similarity, for assessing the potential pipeline bias, which represent a promising starting point for creation of consensus -omics reporting criteria. Various international collaborative working groups, such as International Microbiome and Multi-Omics Standards Alliance (IMMSA), WHO-led microbiome reference reagents working group, and international metaproteomics initiative, are working on making guidelines for meta-omics approaches^159–161^. These efforts are important for the application of multi-omics in clinic and regulatory practices, and the development of functional assays, such as *ex vivo* microbiome assay and intestinal cellular models, to enable efficient quality assessment of microbiota-directed therapeutics (Figure 1).

## 6. Conclusion and perspectives

While the microbiome-directed therapy is promising, its application in real life will significantly challenge whole healthcare systems. Health authorities around the world, in collaboration with clinical professionals, are working on developing novel regulatory pathways for improving their capability in regulating and accessing this type of advanced biotherapeutics. Overall, a shift from the current host- or single pathogen-targeting medicine to more broadly host-microbiome symbiosis-targeting medicine is needed.

To measure the host-microbiome symbiosis, advanced techniques that enable more comprehensive and efficient profiling of the microbiomes are crucial. With the development of enabling technologies, such as NGS and high-resolution MS, multi-omics approaches are becoming more affordable, easily accessible and more informative. It is expected that these advanced -omics approaches can be incorporated into drug development, quality assessment, and even the physio-biochemical examinations for guiding diagnostic and treatment decision. In the meantime, new bioassays that enable the functional assessment of microbiomes, such as *ex vivo* microbiome assay and *in vitro* intestinal models, will also be developed and applied for microbiome investigations. The implementation of these -omics approaches and the anaerobic microbiome assays into the current practices is therefore an important step for enabling safe and efficient delivery of microbiota-directed therapeutics to patients.

To note, although our knowledge on microbiome is markedly expanded in recent years, there remain a lot of unknowns in the field, some of which are fundamental and crucial. In-depth investigations on the mechanisms of host- and drug-microbiome interactions with continuous funding support remain a priority in the coming years.

### List of Abbreviations

**Table:** 

*Abbreviation*	*Definition*
AMPs	Antimicrobial peptides/proteins
AMR	Antimicrobial resistance
Anti-PD-1	Anti – programmed cell death protein 1
ASD	Autism-spectrum disorders
EPS	Exopolysaccharides
EV	Extracellular vesicle
FDA	Food and Drug Administration
FFT	Fecal filtrate transplantation
FMT	Fecal microbiota therapy
FPRA	False positive relative abundance
GI	Gastrointestinal
HuMiX	Human microbial co-culture model
IBD	Inflammatory bowel disease
IBS	Irritable bowel syndrome
iHACS	Intestinal Hemi-Anaerobic Co-culture System
IMMSA	Microbiome and Multi-Omics Standards Alliance
LBPs	Live biotherapeutic products
mAb	Monoclonal antibody
MET	Microbial Ecosystem Therapeutic
MHC	major histocompatibility complex
MIMICS	Mimetic Intestinal Host – Microbe Interaction Coculture System
MS	Mass spectrometry
NGS	Next-generation sequencing
Phe	Phenylalanine
PKU	Phenylketonuria
PTMs	Post-translational modifications
rCDI	Recurrent *Clostridioides difficile* infection
SARS-CoV2	Severe acute respiratory syndrome coronavirus 2
SCFA	Short-chain fatty acid
SHIME	Simulation of the human intestinal microbial ecosystem
SIHUMIx	Simplified human intestinal microbiota
STING	Stimulator of interferon gene
TCR	T cell receptor
WHO	World Health Organization
